# Ascending Aortic Aneurysms: From Pathophysiology to Surgical Repair

**DOI:** 10.3390/jcm14196993

**Published:** 2025-10-02

**Authors:** Waël Oweini, Jalal Jolou, Tornike Sologashvili, Nicolas Murith, Christoph Huber, Mustafa Cikirikcioglu

**Affiliations:** 1 Faculty of Medicine, University of Geneva, 1206 Geneva, Switzerland; 2 Division of Cardiovascular Surgery, Department of Surgery, Geneva University Hospitals (HUG), 1205 Geneva, Switzerland; jalal.jolou@hug.ch (J.J.);

**Keywords:** ascending aorta aneurysm, thoracic aortic disease, surgical thresholds, vascular imaging, genetic aortopathy

## Abstract

The aorta, once viewed as a passive conduit, is now recognized as an active organ crucial for hemodynamic regulation and vascular homeostasis. Thoracic aortic aneurysms (TAAs), particularly those involving the ascending aorta, often remain silent until life-threatening complications such as dissection or rupture occur. Current management primarily relies on aortic diameter criteria, yet up to 60% of type A dissections occur at sizes below the 5.5 cm surgical threshold, revealing the limitations of this approach. This narrative review summarizes recent advances in understanding ascending aortic aneurysms, including insights into their genetic and degenerative mechanisms, the role of novel morphological and hemodynamic markers, and the potential of advanced imaging techniques. It also explores evolving surgical strategies, from conventional open repair, still the gold standard, to minimally invasive and investigational endovascular approaches. By integrating biological, morphological, and clinical factors, emerging strategies aim to move beyond diameter alone toward more personalized risk assessment. This paradigm shift may improve early detection, optimize surgical timing, and ultimately enhance outcomes for patients with ascending aortic aneurysms.

## 1. Introduction

Recent advances in the understanding of aortic function and pathology have led the scientific community to recognize the aorta as a fully fledged organ, owing to its autonomous and vital role. Far from being a passive conduit, the aorta actively contributes to hemodynamic regulation. It plays a key role in mechanosensation, transmits molecular signals, and responds dynamically to hemodynamic stress. This functional complexity is mirrored by a specific pathophysiology, exemplified by distinct disease entities such as aneurysms, dissections, and genetic syndromes like Marfan syndrome [1]. Although this literature review focuses exclusively on the ascending aorta, the segment between the sinotubular junction and the brachiocephalic trunk [2], we will frequently refer in the following sections to thoracic aortic aneurysms (TAAs) in discussions of epidemiology, etiology, and pathophysiology. This is not an oversight but reflects inherent limitations: the aorta lacks sharply demarcated functional compartments, most studies consider TAAs as a whole, and all thoracic segments, except the descending portion, share a common neural crest-derived embryological origin [3].

With regard to aneurysms, these refer to a localized or diffuse enlargement of the three layers of an artery, defined by a diameter that is at least 50% greater than its normal size. In the case of the thoracic aorta, it is considered dilated when the diameter falls between 4.0 cm and 4.4 cm, and is classified as aneurysmal once it reaches or exceeds 4.5 cm [1]. Theoretically, individuals with an aorta larger than 4.5 cm are 6305 times more likely to have aortic dissection [4]. TAAs affect the aortic root or ascending aorta, accounting for approximately 60% of cases. In comparison, 40% involve the descending thoracic aorta, while 10% affect the aortic arch and another 10% extend into the thoracoabdominal segment [5]. Also, TAAs are often referred to as “silent killers” owing to their asymptomatic course in approximately 95% of cases and the potentially fatal consequences of rupture [6]. When rupture occurs, it is associated with a high mortality rate, estimated between 50% and 80% [7]. Even in the absence of rupture, TAAs are associated with a substantial risk of acute aortic syndromes, including acute aortic dissection, intramural hematoma, and penetrating aortic ulcer. This clinical latency constitutes a major barrier to early diagnosis and hinders the implementation of effective screening strategies.

Despite advances in cardiovascular imaging and improvements in surgical techniques, a significant number of acute complications still occur at stages not anticipated by current criteria, particularly aortic diameter, which remains the primary risk indicator. In this context, a deeper understanding of the epidemiological profile, risks factors, pathophysiological mechanisms, etiologies, and morphological determinants of TAAs is essential to refine risk stratification, improve screening strategies, and optimize surgical indications. This literature review aims to synthesize current knowledge concerning ascending aortic aneurysms, highlighting the limitations of existing criteria and exploring emerging approaches for a more personalized strategy.

## 2. Methods

A comprehensive literature review was performed to synthesize current knowledge on ascending aortic aneurysms. Three major databases: PubMed, Cochrane Library, and Google Scholar, were systematically searched for articles published between 2015 and 2025. The search strategy combined keywords such as “ascending aortic aneurysm,” “thoracic aortic disease,” “genetic aortopathy,” “aortic surgery,” and “surgical thresholds.” A total of 91 articles were ultimately included, selected for their relevance to key domains, such as epidemiology, pathophysiology, etiology, surgical indications, and evolving management strategies. This approach ensured a broad yet focused overview, capturing both clinical and molecular insights to provide a robust foundation for the review.

## 3. Epidemiology

The main limitation of available epidemiological data on TAAs stems from two primary factors. First, most TAAs remain asymptomatic until the occurrence of an acute event or are diagnosed incidentally, thereby limiting detection in the general population [8]. Second, discrepancies in measurement criteria across guidelines contribute to the scarcity and heterogeneity of epidemiological data, making comparisons challenging [1]. As a result, reported incidence rates vary widely across studies, ranging from 5.3 to 13.8 cases per 100,000 person-years [6,9,10].

In their study, Gouveia and Melo et al. estimated the incidence of TAAs as 5.3 cases per 100,000 person-years. The pooled overall prevalence was calculated at 0.16% using a random-effects model based on 22 population-based studies. When stratified by study type, prevalence was 0.07% in studies based on clinical screening of living individuals and 0.76% in autopsy studies, highlighting methodological differences in case detection [9]. These findings highlight two important points. First, there is a significant discrepancy, either an overestimation or an underestimation, between screening and post-mortem studies. Second, this discrepancy helps explain the extent of underdiagnosis in the general population. For instance, in a city such as Geneva, which has approximately 200,000 inhabitants, the estimated number of individuals with a TAA (diagnosed or not) would be around 320, with approximately 11 new cases emerging each year. However, if one considers only the prevalence derived from screening studies (0.07%), this would imply that nearly 180 individuals with a TAA, representing 56% of cases, would remain undiagnosed and not treated.

Epidemiological patterns of TAAs are also modulated by differences related to sex and ethnicity. While some population-based studies suggest similar prevalence between men and women [11], others report a twofold higher frequency in men, with women exhibiting more aggressive disease profiles, including faster aneurysm growth and higher risk of complications [12]. Additionally, women are typically diagnosed later, often a decade older [11]. Ethnic differences are also pronounced: for example, Chinese individuals show larger ascending aortic diameters than Caucasians, while Vietnamese and Malaysian populations demonstrate accelerated aortic growth [13,14,15].

## 4. Etiology and Risks Factors

TAAs encompass a range of etiologies that can be broadly classified according to their anatomical location relative to the ligamentum arteriosum. Distal to this landmark, TAAs are predominantly atherosclerotic in nature and share traditional cardiovascular risk factors, such as male sex, advanced age, hypertension, chronic obstructive pulmonary disease, high non-HDL levels, low HDL levels, and smoking [16,17].

Concerning proximal aneurysms, these are most commonly associated with cystic medial degeneration, a degenerative process of the aortic media characterized by loss of smooth muscle cells, elastic fiber fragmentation, and accumulation of proteoglycans. This histological pattern is classically observed in heritable thoracic aortic diseases (HTADs), particularly in syndromic forms such as Marfan, Loeys–Dietz, or vascular Ehlers–Danlos syndromes. Non-syndromic HTADs and familial aggregations without overt extra-aortic features also share this underlying mechanism [16]. Although genetic factors predominate in proximal aneurysms, age-related degeneration may also contribute to the disease process, particularly in patients without identifiable mutations. Other etiologies include non-infectious inflammatory diseases (e.g., giant cell arteritis, Takayasu arteritis, systemic lupus erythematosus), congenital anomalies such as bicuspid aortic valve (BAV), and, more rarely, infectious causes, including mycotic aneurysms, syphilis, or endocarditis [16]. Approximately 30% of TAAs are attributable to single-gene mutations, most of which follow an autosomal dominant inheritance pattern; around 5% of these are syndromic, while 21% are non-syndromic [16,18]. The remaining 70% are considered “sporadic” in the absence of identifiable genetic or syndromic associations. These causes differ notably in the age of clinical onset: genetic forms manifest early (in young individuals), BAV-associated forms appear in middle age, and “sporadic” forms primarily affect older adults.

While the literature still refers to “sporadic” or “degenerative” TAAs, these terms are increasingly challenged by genetic findings. The expanding use of next-generation sequencing has revealed that many apparently sporadic aneurysms may have undetected genetic origins. Supporting this, up to one-third of first-degree relatives of patients without an identified pathogenic variant also exhibit thoracic aortic dilation. Consequently, distinguishing truly sporadic from heritable forms remains challenging, and current practice favors categorizing patients based on whether a pathogenic variant has been identified or not [1].

On another note, some data suggest that type 2 diabetes may exert a paradoxically protective effect against TAA development. Koba et al. and Zhang et al. reported a 22% decrease in the prevalence of aortic aneurysms among diabetic patients and reduced aortic mortality. This inverse association could be explained by mechanisms such as glycation of aortic wall proteins, resulting in increased stiffness, or the anti-inflammatory effects of certain antidiabetic drugs like metformin [17,19]. However, further studies are required to confirm these hypotheses and elucidate the underlying mechanisms.

## 5. Pathophysiology

### 5.1. Normal Aortic Structure and Function

The ascending aorta is the main conduit for blood flow and the largest artery in the human body. Its wall comprises three layers: the intima, the elastic media, and the adventitia. The intima is lined with endothelial cells (ECs), which form the internal lining of all blood vessels. These cells serve both as a selectively permeable barrier between the blood and surrounding tissues and as primary “sensors of flow-induced wall shear stress,” enabling them to influence the contractility of vascular smooth muscle cells (vSMCs) and thus modulate arterial diameter [20]. In a healthy ascending aorta, the elastic media is 0.5 to 2 mm thick and accounts for approximately 80% of the aortic wall thickness [21]. It primarily contains vSMCs, fibroblasts (FBs), and an extracellular matrix (ECM) composed of elastin, collagen, glycosaminoglycans, and other components [20]. The media consists of more than 50 concentric layers alternating between elastic lamellae and vSMCs. The adventitia, in turn, is primarily rich in collagen, vasa vasorum, nerve fibers, lymphatic vessels, and adipocytes [21].

This structure gives the aorta both compliance and resilience. Given the pulsatile nature of blood flow, compliance is ensured by the numerous elastic lamellae, whose number gradually decreases along the length of the aorta. This property allows the ascending aorta to store energy during systole and release it during diastole, propelling blood distally, a phenomenon known as the Windkessel effect. Resilience, on the other hand, is conferred by collagen fibers, which act as a protective sheath preventing excessive distension of the aorta and preserving the integrity of the elastic fibers and microfibrillar–cellular connections within the media [21].

From a structural perspective, the contractile function of vSMCs depends on a specialized structure known as the elastin-contractile unit. Mechanical signals originating from ECs are transmitted through the extracellular elastin network to focal adhesions, also referred to as dense plaques, on the vSMC plasma membrane. These signals are then relayed intracellularly by anchoring and actin-binding proteins, ultimately leading to vSMC contraction [22]. Any imbalance or structural defect within the aortic wall leads to weakening of the vessel and progressive dilatation.

### 5.2. Vascular Smooth Muscle Cells

In addition to their contractile role, vSMCs maintain aortic wall elasticity and synthesize key ECM components such as collagen and elastin [3]. Apoptotic loss of vSMCs is an early hallmark of aneurysm formation, reducing structural integrity and ECM regenerative capacity, thereby weakening the wall and promoting dilation [3]. As the aorta dilates, per Laplace’s law, wall stress increases, amplifying the risk of dissection or rupture [5]. vSMC survival depends on stable anchorage to the ECM and preservation of the mechanical architecture (“tensegrity”). Disruption of fibronectin, integrins, the cytoskeleton, or focal adhesion complexes (FAK, Src, talin) impairs mechanotransduction, leading to anoikis, apoptosis induced by ECM detachment [18]. Thus, genetic mutations, inflammation, or proteolytic degradation destabilize vSMCs and contribute to wall weakening.

Under physiological conditions, vSMCs exhibit a contractile phenotype with spindle morphology, low proliferation, and expression of α-SMA, calponin 1, SM22α, and SMMHC [23,24]. In response to injury, stress, or hypertension, they switch to a synthetic phenotype, enabling proliferation, migration, and proteolytic enzyme secretion [23]. While essential for repair, sustained switching promotes ECM degradation, excessive protease activity, and apoptosis, driving aneurysm progression [23].

### 5.3. Extracellular Matrix Degradation

As previously discussed, in response to injury, vSMCs excessively secrete proteolytic enzymes, such as MMPs, ADAMs, ADAMTS, cathepsins, and fibrinolytic activators, thereby exacerbating wall degeneration and fostering aneurysm development. Increased expression of MMP-1, MMP-2, MMP-9, ADAM-17, and ADAMTS-1/-4 has been consistently reported in human abdominal aortic aneurysms and, similarly, in aneurysmal tissue of TAAs [3]. Cathepsins B, D, G, L, and S also contribute to elastin fragmentation, apoptosis, and inflammation [3].

To illustrate this point, Chung et al. demonstrated in a murine model of Marfan syndrome that doxycycline reduced TAA progression more effectively than atenolol by inhibiting MMP-2/-9, preserving elastin and vSMC viability, highlighting MMP inhibition as a possible therapeutic target [25].

### 5.4. Aortic Wall Inflammation

Pisano et al. highlighted the central role of inflammation in TAA pathophysiology, challenging the traditional view of these lesions as purely degenerative and non-inflammatory. Their study demonstrated macrophage and T-lymphocyte infiltration in “sporadic” TAAs, along with Fas/FasL-mediated vSMC apoptosis and upregulation of p53, Bax, and TUNEL markers, suggesting that inflammation-driven cellular loss may precede dissection independently of aortic diameter [3,26].

### 5.5. Outward Convection and TGF-β Pathway

The ~100 mmHg transmural gradient between the aortic lumen and adventitia drives outward convection, enabling the transport of soluble and macromolecular plasma components across the aortic wall, including albumin, prothrombin, and plasminogen [18]. Once activated locally, these proteins can detach vSMCs from the ECM, trigger protease activation, and release transforming growth factor-β (TGF-β) from its matrix reservoirs. TGF-β, secreted by vSMCs as an ECM-bound latent complex, is also activated by plasmin, MMPs, or integrin-mediated tension [18]. This pathway regulates vSMC phenotype, ECM synthesis, and inflammation, thus maintaining aortic wall stability. Genetic mutations or pathological protease activity disrupts TGF-β signaling, leading to vSMC dysfunction, aberrant ECM remodeling, and chronic inflammation, ultimately increasing susceptibility to dilation, rupture, and dissection [27].

### 5.6. Role of Aging

Aging reduces elastin, alters collagen, and depletes vSMCs, impairing compliance and integrity. Senescence marked by telomere shortening, decreased telomerase activity, and increased expression of markers such as p16INK4a and p19ARF altered glycosaminoglycan metabolism, and mitochondrial dysfunction activates innate inflammation (TLR9, inflammasome, STING), driving aneurysm progression [28].

### 5.7. Bicuspid Aortic Valve and Altered Hemodynamics

BAV affects 1–2% of adults, nearly half of whom develop aneurysms, with an eightfold higher dissection risk than the general population [29]. Moreover, aortic growth is faster in BAV patients (0.2–0.6 mm/year) than in those with a tricuspid aortic valve (0.1–0.3 mm/year) [1]. The location of the aneurysm is influenced by the type of cusp fusion: right–left cusp fusion (70–80%) redirects blood flow toward the anterior proximal ascending aorta, whereas right–noncoronary cusp fusion deflects flow toward more distal segments and the aortic arch [30,31]. Pasta et al. showed BAV patients exhibit elevated wall shear stress (WSS) and time-averaged WSS (TAWSS) correlating with MMP-1/-2, TIMP-1, and microRNAs (miR-26a, miR-320a), highlighting a specific biological response to altered mechanical stress and linking altered flow to ECM degradation and remodeling [32].

### 5.8. Genetic Factors

The pathogenesis of ascending aortic aneurysms is frequently influenced by genetic factors, which affect signaling pathways or proteins involved in the contractile apparatus of vSMCs as well as components of the ECM. These mutations disrupt vSMC contractility and their interactions with the ECM, weakening the aortic wall. vSMC dysfunction leads to impaired contraction, aberrant environmental sensing, abnormal cell proliferation, and ECM degradation. These changes contribute to pathological remodeling of the aortic wall, increasing the risk of dilation, rupture, or dissection [33]. Table 1 and Table 2 provide examples of syndromic and non-syndromic HTAD associated with ascending aortic aneurysm development.

## 6. Surgical Indications

### 6.1. Aortic Diameter

Surgical indication for ascending aortic aneurysms primarily relies on the measurement of maximum aortic diameter. In asymptomatic patients with ascending aortic aneurysms, surgery is uniformly recommended once the maximal diameter reaches 5.5 cm, according to the 2022 ACC/AHA (COR I), 2024 EACTS/STS (Class I, Level B), and 2024 ESC (Class I, Level B) guidelines.

In certain clinical contexts, all three guidelines allow re-evaluation of the surgical threshold, with slightly lower classes of recommendation. For instance, in asymptomatic patients with a tricuspid aortic valve, surgery may be considered at 5.0 cm, according to ACC/AHA (COR 2a), EACTS/STS (Class IIa, Level C), and ESC 2024 (Class IIa, Level C). Furthermore, when aortic valve replacement is simultaneously required, surgical intervention may be considered at 4.5 cm under the same respective recommendations (ACC/AHA: COR 2a; EACTS/STS: IIa C; ESC 2024: IIa, Level C).

Significant differences arise regarding threshold reductions in the presence of additional risk factors or genetic conditions. The EACTS/STS and the ESC 2024 guidelines adopt a more individualized approach, lowering the threshold to 5 cm in low-risk patients when one or more risk modifiers are present, such as age <50 years, short stature (<1.68 m), rapid aortic growth (>3 mm/year), ascending aortic length >11 cm, or a family history of acute aortic syndromes. Moreover, they also advocate for earlier intervention in patients planning pregnancy. The ACC/AHA guidelines adopt a generally more conservative approach, maintaining the 5.5 cm threshold as standard. However, they allow for elective surgery at 5.0 cm in experienced centers (COR 2a). Surgery is also indicated if the growth rate reaches ≥0.3 cm/year over two consecutive years or ≥0.5 cm within a single year (COR 1).

Regarding genetic syndromes, all three guidelines define precise surgical thresholds for Marfan and Loeys–Dietz syndromes. In Marfan syndrome, intervention is advised at 5.0 cm, or 4.5 cm in the presence of risk factors. In Loeys–Dietz syndrome, the threshold may be lowered to 4.0 cm, depending on the genetic variant, growth rate, and individual risk profile, ideally assessed within a multidisciplinary aortic team. Although all societies provide specific thresholds for heritable thoracic aortic diseases, only the ACC/AHA guidelines explicitly recommend prophylactic surgery at ≥4.0 cm for women with connective tissue disorders who plan pregnancy.

This harmonization of thresholds across major international guidelines paves the way for presenting a summary table (Table 3) [1,34,35,36].

The concept of the “hinge point,” was originally defined by a landmark study by Coady et al. in 1997 based on a cohort of 230 patients. The study identified a critical median diameter of 6 cm, beyond which the risk of dissection or rupture increased by 32.1%. The authors noted that, because 6 cm represents a median, approximately half of all patients would have already experienced a life-threatening complication if surgery were delayed until that point. As a result, a preventive threshold of 5.5 cm was proposed to reduce acute events while balancing operative risk [37]. Subsequent studies identified hinge points at even lower diameters, 5.25 cm and 5.5 cm, beyond which complication risks increased significantly [38]. These findings have prompted the hypothesis that lowering the surgical threshold to 5.0 cm could improve complication prevention. Perez et al. evaluated this approach, showing that 69.1% of aortic dissections occurred below 5.5 cm and that lowering the threshold could prevent 29.3% of unexpected dissections, amounting to a 42% overall reduction in type A dissections occurring outside current guideline recommendations [39]. It is important to note that in this study, aortic measurements were taken after dissection. However, it has been demonstrated that acute dissection increases the aortic diameter by ~30%, equivalent to a 7–8 mm enlargement [40,41]. This supports the idea that dissections often occur at diameters below 5.0 cm.

To strengthen the case for a “left-shifted threshold,” Saeyeldin et al. conducted a clinical study of 781 patients, comparing outcomes between a group that underwent prophylactic surgery at 5.0 cm and a group that refused surgery. Their findings showed that mortality and risk of dissection/rupture were higher among patients who declined surgery than the 30-day operative mortality. They concluded that prophylactic surgery at 5.0 cm is justified and proposed a decision algorithm to guide surgical indications, recommending surgery for all patients with an ascending aortic aneurysm ≥5.0 cm, as well as for those measuring 4.0–4.9 cm when associated with chest pain, strong family history, severe connective tissue disease, or bicuspid aortic valve. In operated patients, surgery carries a 30-day mortality of ~1%, compared with a ≤12% risk of aortic death and 13% risk of an aortic event in non-operated patients, whereas medical management is advised for 4.0–4.9 cm aneurysms without these additional risk factors (aortic death ≤ 0.6%, aortic event 2%) [38]. These observations are corroborated by data from the International Registry of Aortic Dissection (IRAD), which show that 60% of type A dissections occur at diameters < 5.5 cm and 40% occur at diameters < 5.0 cm, a phenomenon known as the “Aortic Size Paradox” [42].

### 6.2. Imaging-Based Limitations

The most commonly used imaging modalities to measure and monitor aortic aneurysms include computed tomography (CT), magnetic resonance imaging (MRI), and echocardiography. However, inherent methodological and resolution differences among these techniques can yield variable measurements. Furthermore, because these measurements are often performed by different operators, inter-observer variability is introduced, and errors can arise, especially when measurements are taken on oblique planes. There is also no universal consensus on the most appropriate method of measuring aortic diameter, such as the optimal level of measurement or whether to include the vessel wall. The ACC/AHA and the ESC guidelines recommend an inner-edge-to-inner-edge measurement for greater consistency and reproducibility, while the EACTS/STS guidelines use an outer-edge-to-outer-edge approach, which tends to result in larger values [34]. Each modality also has specific limitations: incomplete visualization of the ascending aorta by echocardiography, differences in measurement with or without contrast in CT imaging, and motion artifacts in CT during systole or respiration. Measurement variability can reach several millimeters, which has significant implications for clinical decision-making and patient follow-up [43]. Table 4 provides a concise overview of the main imaging modalities used in clinical practice, highlighting their advantages, limitations, and recommended follow-up intervals.

Taken together, recent data increasingly suggest that aortic diameter, at least as it is currently used, is an imperfect predictor for surgical decision-making. This has led to growing interest in lowering the surgical threshold (e.g., to 5.0 cm or lower) and in exploring additional risk markers, which are detailed in the next section.

### 6.3. Other Surgical Indicators

It is essential to recognize that having an aortic diameter above the surgical threshold does not necessarily predict acute complications, just as surgical intervention is not without risk. The key challenge lies in identifying measurable parameters that can predict complications more accurately while minimizing unnecessary surgery. As previously discussed, aortic diameter alone provides an incomplete risk estimate. Consequently, research has turned toward alternative metrics. Although none of these have yet been incorporated into international guidelines, they represent promising avenues for future practice.

#### 6.3.1. Aortic Length and Volume

Advances in 3D vascular imaging technology now allow precise assessments of the aorta’s geometry, including length and volume [44]. Building on prior work identifying critical diameter thresholds, Wu et al. investigated hinge points for ascending aortic length. Aortic length was defined as the centerline distance from the aortic annulus to the origin of the brachiocephalic trunk. They identified two significant thresholds, 11 cm and 13 cm, above which the risk of acute complications increased markedly. Specifically, a length ≥ 13 cm was associated with a 32% higher risk compared to a length < 7 cm. Among patients who had experienced a dissection, 44 had pre-dissection diameters < 5.5 cm. Of those, 31 patients (70.4%) had ascending aortic lengths ≥ 11 cm. These results highlight the importance of integrating aortic length, in addition to diameter, into risk stratification, as is done by the EACTS. Wu et al. even proposed that 11 cm may represent a relevant threshold for surgical indication [45]. Importantly, aortic length appears to remain stable after dissection, with only a modest increase of ~2.7% (similar to other studies reporting increases of around 5.4%) [45,46]. This suggests that it may provide a more reliable basis for determining rupture thresholds in post-mortem studies.

Moreover, in a retrospective study of 477 patients, Heuts et al. assessed the diagnostic performance of ascending aortic length and volume to predict type A dissection. Two groups were compared: one with dissection and one with aneurysm but no dissection. The pre-dissection aortic length was significantly longer in the dissection group (90 ± 16 mm) than in controls (84 ± 9 mm). They then evaluated the sensitivity of each parameter and combinations. The use of aortic length instead of diameter increased sensitivity from 4% to 28%. When length and diameter were combined into a volume-based measure, the sensitivity was lower but reproducibility was higher, which is a key advantage in clinical follow-up [44,47]. It is also noteworthy that ascending aortic length correlates with age in healthy individuals, increasing by approximately 3 mm per decade [48].

In a similar study, Krüger et al. reported concordant results. The switch from diameter-based to volume-based assessment increased diagnostic sensitivity from 5.3% to 15.8%. Notably, they also examined specificity: false positives were significantly reduced from 5.4% (diameter-based) to just 0.6% (volume-based). These findings suggest that combining aortic length and diameter offers a superior risk stratification strategy compared to diameter alone [49].

It is important to note that Heuts et al. and Krüger et al. did not establish a specific threshold for aortic volume. Instead, they assessed sensitivity and specificity based on two parameters, length and diameter, used individually and in combination. While no definitive volume cutoff was defined, their findings underscore the potential utility of volumetric assessment as an emerging parameter to improve risk stratification beyond traditional diameter-based criteria [44,49].

#### 6.3.2. Vessel Wall Inflammation

As previously mentioned, inflammation of the aortic wall may play a key role in ascending aortic aneurysm development. In this context, positron emission tomography (PET) using fluorodeoxyglucose (FDG), a glucose analog taken up by metabolically active inflammatory cells, has emerged as a particularly promising cardiovascular imaging tool [50].

The value of FDG-PET/CT for predicting aortic events has been most thoroughly studied in abdominal aortic aneurysms. Elevated FDG uptake (SUVmax > 2.5) was frequently observed and correlated with histological signs of inflammation, including macrophage and T-cell infiltration, decreased collagen content, and vSMC loss [51]. Sakalihasan et al. also reported that the rate of acute complications was 67% in FDG-positive patients versus 20% in those with negative scans [52]. However, interindividual variability in FDG uptake complicates interpretation and limits the technique’s predictive value in clinical practice [53]. Despite these limitations, the method remains promising and may also be applicable to ascending aortic aneurysms. This is illustrated in a case series by Tahara et al., in which four patients who later developed type A dissection had previously undergone FDG-PET/CT (within 5 years) for unrelated reasons. Abnormal FDG uptake, either focal or segmental, was noted in all four patients, specifically in the region where the dissection eventually occurred. These findings suggest that chronic inflammation or subclinical wall remodeling may precede dissection and that FDG-PET/CT could potentially identify high-risk patients before complications arise [54]. Similar observations were reported by Simsek et al. in another series of case reports [55].

Beyond FDG, a new generation of PET radiotracers has been developed to visualize specific molecular processes involved in aneurysm progression. Although most research has so far focused on abdominal aortic aneurysms, these tracers offer promising potential for application in thoracic aortic aneurysms. They include agents targeting matrix metalloproteinases (MMPs), ^18^F-sodium fluoride (^18^F-NaF) for detecting microcalcifications, and macrophage-targeted nanoparticles labeled with fluorine-18, which enable more precise visualization of aortic wall inflammation [56]. Notably, ^18^F-NaF uptake has been reported to be up to 30% higher in rapidly expanding aneurysms compared with stable ones, underlining its potential to identify regions of active remodeling. Similarly, macrophage-targeted nanoparticles labeled with fluorine-18 demonstrated nearly a 50% increase in signal-to-noise ratio compared with conventional imaging, allowing for finer detection of inflammatory hotspots [56]. Experimental MMP-targeted tracers also exhibit a direct correlation between PET signal intensity and plasma levels of MMP, supporting their role as biomarkers of extracellular matrix degradation [56].

#### 6.3.3. Hemodynamics

4D Flow MRI (or 4D MRI) is an advanced imaging technology that allows the analysis of blood flow in three spatial dimensions while incorporating the temporal dimension. This technique captures the movement of blood through vessels over specific time intervals, providing a dynamic and comprehensive depiction of blood circulation. 4D Flow MRI enables precise measurement of hemodynamic parameters such as blood flow velocity, wall shear stress (WSS), and flow jet displacement. It thus offers detailed insights into blood flow mechanics, allowing for the detection of anomalies such as turbulence or stagnation zones [57]. The study by Mohammad Al-Rawi et al. highlights the critical role of several hemodynamic parameters in predicting aortic dissections. Among these, WSS emerges as a key indicator, as abnormally high values can weaken the aortic wall, making it more prone to tearing. In their computational modeling, zones with WSS values between 2 and 6 Pa were observed in dilated regions of the ascending aorta, indicating areas of medial wall degradation. Conversely, regions at the inlet and outlet of the aorta demonstrated much higher WSS values, exceeding 5 Pa and reaching up to 10 Pa post-dissection, suggesting a significant mechanical load in those zones. Time-averaged wall shear stress (TAWSS), which integrates WSS over the cardiac cycle, reached up to 5 Pa in the pre-dissection state and as high as 10 Pa in post-dissection models. These elevated values were particularly notable at the arch and in the vicinity of supra-aortic vessels, underlining their predictive relevance. The oscillatory shear index (OSI), which quantifies the directional changes of shear stress, ranged from 0 to 0.5, with higher values reflecting regions of disturbed, multidirectional flow, frequently coinciding with the origin sites of dissections. Similarly, the relative residence time (RRT), which captures the degree of blood flow stagnation near the vessel wall, was markedly elevated in high-risk zones, reinforcing its association with endothelial dysfunction and inflammatory processes [58]. Additionally, other studies using fluid–structure interaction models suggest that regions with TAWSS values below 0.2 Pa are prone to thrombus formation and pathological remodeling, further emphasizing the importance of these hemodynamic metrics in risk stratification [59].

Together, these findings underscore the predictive utility of WSS, TAWSS, OSI, and RRT in identifying aortic segments vulnerable to dissection. Their integration into advanced 3D morphometric and computational flow models may ultimately enhance clinical decision-making by supplementing traditional diameter-based assessments.

#### 6.3.4. Aortic Stiffness

In a recent study by Fortunato et al., the aortic stiffness index, calculated non-invasively via echocardiography, was shown to be a robust predictor of type A dissection in patients with ascending aortic dilation. Compared to aneurysmal patients without dissection, those who experienced dissection had significantly higher stiffness indices. A value above 5 predicted dissections with a sensitivity of 91% and specificity of 78%, outperforming conventional anatomical indices like maximum diameter. The stiffness index was also positively correlated with peak longitudinal wall stress, supporting a pathophysiological link between aortic rigidity and dissection risk.

Formula for Aortic Stiffness Index:

Stiffness Index=(ln(SBP/DBP))/(((AoS−AoD)/AoD))

where:

- *SBP* = Systolic blood pressure (measured at the arm),
- *DBP* = Diastolic blood pressure,
- *AoS* = Ascending aortic diameter in systole (early T wave on ECG),
- *AoD* = Ascending aortic diameter in diastole (peak R wave on ECG).

These findings suggest that incorporating aortic stiffness into risk stratification could improve surgical decision-making, especially in patients whose diameter remains below guideline thresholds [60].

#### 6.3.5. Biological Markers

Beyond morphometric and hemodynamic parameters, circulating biomarkers are emerging as valuable tools for refining risk stratification in TAAs. Several studies have demonstrated associations between plasma markers and disease activity, including matrix metalloproteinases (MMPs), tissue inhibitors of metalloproteinases (TIMPs), inflammatory cytokines, and microRNA (miRNA) signatures. These circulating miRNAs, MMPs, TIMPs, and inflammatory cytokines likely originate from endothelial cells and vascular smooth muscle cells undergoing stress, phenotypic modulation, or apoptosis, and are released into the bloodstream via exosomes or following cell injury, making them attractive non-invasive biomarkers for early disease detection and monitoring [61].

Several circulating biomarkers show strong quantitative associations with aortic hemodynamics and TAA progression. In their study, Pasta et al. analyzed a cohort of 125 patients, and time-averaged wall shear stress (TAWSS) correlated with MMP-1, MMP-2, and TIMP-1 activity (e.g., MMP-1 vs. TAWSS R = 0.52; *p* < 0.001), while in BAV patients, aortic wall strain was associated with miR-26a (R = 0.55; *p* = 0.041) and miR-320a (R = 0.69; *p* < 0.001). A model combining WSS, MMP-1, TIMP-1, and MMP-12 achieved an AUC = 0.898 for predicting surgical indication, suggesting that altered wall shear stress drives a specific proteolytic and miRNA response [32]. Complementary evidence from Ngo Bilong Ekedi et al. further supports the diagnostic and prognostic value of circulating miRNAs. In a case–control study including 38 patients with isolated TAAs, 67 with coronary atherosclerosis, 22 with combined disease, and 17 healthy controls, next-generation sequencing of plasma samples revealed a distinct miRNA signature in the TAAs. Significant upregulation of miR-21-5p, miR-29b-3p/-5p, miR-126-3p/-5p, miR-181b-5p, miR-92a-3p, and miR-145-5p was observed (*p*-values ranging from 10^−5^ to 0.0014), with several miRNAs achieving excellent discriminative performance for TAAs versus controls (AUC > 0.80) [61].

In line with these findings, Thijssen et al. highlighted additional circulating markers for thoracic aortic disease, including MMP-2, MMP-9, TIMP-1, TIMP-2, and inflammatory cytokines (TGF-β, IL-6, TNF-α), which reflect ECM remodeling and medial degeneration. They also emphasize the clinical relevance of miR-21, miR-29b, and miR-145, which regulate SMC phenotype, ECM synthesis, and apoptosis, and show strong discriminative performance for TAAs (AUC > 0.80) [62].

Recent evidence from Li et al. adds another layer by identifying matricellular proteins (MCPs), including osteopontin, thrombospondins (TSP-1, TSP-2), tenascin-C, periostin, and SPARC, as mechanistic contributors to TAAs and as potential circulating biomarkers. In aneurysmal tissue, OPN and TSP-1 were upregulated by two- to threefold compared with healthy aorta (*p* < 0.01), and plasma levels of OPN and tenascin-C were significantly higher in TAA patients versus controls (median 85 vs. 42 ng/mL for OPN; *p* < 0.001). MCP expression correlated with aneurysm diameter (R = 0.48–0.62; *p* < 0.01) and growth rate, and in ex vivo studies, MCPs enhanced MMP-2 and MMP-9 activity by approximately 1.8–2-fold, promoting ECM degradation [63].

In conclusion, these findings underscore the promising potential of circulating biomarkers, including MMPs, TIMPs, inflammatory cytokines, miRNAs, and matricellular proteins, for improving risk stratification in thoracic aortic aneurysms. They provide mechanistic insights into ECM remodeling, inflammation, and cellular responses, and several show strong discriminative performance for TAA progression. However, despite these encouraging results, these biomarkers are not yet integrated into routine clinical practice. Further large-scale, prospective, and multicenter studies are needed to validate their predictive accuracy, determine standardized thresholds, and assess their impact on patient management before they can be widely applied in everyday clinical care.

#### 6.3.6. Integration of Artificial Intelligence and Machine Learning

Recent work by Hahn et al. demonstrates that artificial intelligence (AI) and machine learning (ML), especially deep learning (DL) models, significantly enhance the extraction of imaging-derived radiomic features from CT and MRI scans in aortic disease. These tools improve segmentation accuracy of the ascending aorta, reduce inter-observer variability, and enable automated quantification of diameter, length, and volume. In comparative studies, DL-based segmentation achieved a Dice similarity coefficient (DSC) of 0.92–0.95 versus manual annotations, outperforming traditional thresholding or region-growing methods (DSC 0.85–0.88).

ML models have also been applied to predict high-risk complications. Using retrospective datasets of 1200 thoracic aortic scans, a DL-based approach predicted dissection or rupture within 12 months, with an area under the curve (AUC) of 0.87–0.91, compared with 0.72–0.78 for diameter-based risk alone. Incorporating flow-derived features from 4D MRI, such as wall shear stress (WSS) and oscillatory shear index (OSI), further improved predictive performance (AUC = 0.92). These results highlight that AI and ML not only automate and standardize imaging measurements but also integrate hemodynamic and morphometric data for superior risk stratification [64].

Building on these imaging-derived approaches, the integration of genetic information through AI further enhances risk stratification in thoracic aortic disease. Pirruccello et al. applied deep learning to cardiac MRI data from the UK Biobank, enabling high-throughput phenotyping of the thoracic aorta and the identification of 82 loci associated with ascending aortic diameter and 47 with descending aortic diameter, of which 14 were shared between segments. By combining these loci into a polygenic score, they showed that individuals with higher scores were significantly more likely to develop thoracic aortic aneurysms (hazard ratio 1.43 per standard deviation; 95% CI: 1.32–1.54; *p* = 3.3 × 10^−20^). These findings suggest that polygenic scores could, in the future, be used to identify high-risk individuals and guide personalized monitoring and preventive strategies, providing an additional layer of risk stratification beyond traditional diameter and flow-based assessment [65].

These results are promising, showing that AI- and ML-based approaches have the potential to improve risk stratification and clinical decision-making. Nevertheless, their integration into routine practice is still lacking, as their application remains mostly confined to research. Robust prospective, multicenter studies are required to confirm their predictive value, evaluate their effect on clinical outcomes, and define standardized implementation strategies.

#### 6.3.7. Conclusion on Surgical Risk Markers

Aortic diameter remains the primary criterion used to assess the risk of major aortic events. However, its limitations as a standalone predictor are now well established, prompting efforts to identify alternative or complementary indicators. Despite promising advances in the field, none of the emerging markers, whether imaging markers or biological markers discussed in the section on pathophysiology, have yet been incorporated into international guidelines. This is partly due to a lack of large-scale validation studies. Notably, many emerging strategies still apply fixed thresholds across all patients, without accounting for individual variability.

## 7. Surgical Technique

### 7.1. Standard Surgical Management

Open surgical repair remains the gold standard for the treatment of ascending aorta aneurysms [5]. The extent of resection is guided by the aneurysm’s location and underlying etiology. While supracoronary ascending aortic replacement may suffice in isolated ascending aorta aneurysms, more extensive interventions involving the aortic root and arch are often required in patients with connective tissue disorders or a strong familial predisposition to dissection or rupture [16].

Standard surgical repair of tubular ascending aortic aneurysms typically begins with a median sternotomy, allowing optimal exposure of the ascending aorta. After systemic heparinization, cardiopulmonary bypass (CPB) is usually established via central cannulation, most often through the right atrium and distal ascending aorta, although peripheral cannulation may be required if central access is contraindicated. Left ventricular venting is routinely used to facilitate cardiac decompression, and myocardial protection is provided using either cold blood or crystalloid cardioplegia, delivered antegrade through the coronary ostia or retrograde via the coronary sinus [66]. For aneurysms confined to the proximal ascending aorta and well separated from the arch, a standard cross-clamp and resection technique is preferred. In this approach, approximately 2 cm of the distal ascending aorta is preserved, with the Dacron graft anastomosed proximally at the sinotubular junction and distally just before the aortic arch. However, borderline distal ascending aortic aneurysms remain controversial, as the cross-clamping technique typically leaves approximately 2 cm of residual aneurysmal tissue. This residual segment may result in graft mismatch and progressive dilation of the adjacent arch, ultimately contributing to aneurysm recurrence rates estimated at 10–20% over mid- to long-term follow-up [67].

### 7.2. General Mortality and Complications

Despite the technical aspect of this procedure, outcomes following replacement of the ascending aorta appear favorable. In a study by Yamabe et al., isolated ascending aortic replacement was associated with low in-hospital mortality and stroke rates (both 1.9%), and a total complication rate of 11.5%, including death, stroke, major bleeding, renal failure, respiratory insufficiency, shock, sternal infection, or permanent pacemaker implantation, suggesting a high degree of procedural safety. However, proximal extension of the aneurysm was associated with a threefold increase in mortality risk, unlike distal extension, which showed no such association. Consistent with these findings, Mori and colleagues analyzed 53,559 proximal thoracic aortic aneurysm surgeries performed electively between 2011 and 2016 across 1045 centers in the United States, reporting a similarly low in-hospital mortality rate of 2.2% [68].

Institutional case volume also appears to be a key determinant of surgical outcomes. Nam et al. demonstrated that surgical volume is a critical determinant, reporting in a nationwide cohort of 4867 thoracic aorta replacements that low-volume centers (≤5 cases/year) had a markedly higher in-hospital mortality rate (21.9%) compared to high-volume centers (≥50 cases/year, 8.6%), corresponding to a 3.1-fold increased adjusted risk of death [69]. Similarly, Mori et al. identified an inflection point at approximately 20–25 cases per year, beyond which operative mortality decreased substantially, by a factor of 2.5 [68].

Despite the surgical simplicity of tubular ascending aorta replacement, it is still important to emphasize the challenges it can present. As a matter of fact, favorable perioperative results may not fully translate into patient well-being. In a prospective study by Hamiko et al., although surgical outcomes were acceptable, quality of life, measured using the SF-36 questionnaire, was significantly impaired compared to the general population. Both physical (PCS: 41.1 vs. 48.4) and mental (MCS: 42.1 vs. 50.9) component scores were markedly lower (*p* < 0.001), with mental health scores notably worse than those reported in myocardial infarction or cancer cohorts [70]. This prompts reflection on whether, despite favorable perioperative outcomes, the goal of truly enhancing patients’ long-term well-being and quality of life is consistently achieved. Nevertheless, further large-scale studies are warranted to substantiate and refine these conclusions.

On the other hand, certain surgery-specific factors may play a role in mid and long-term outcomes. Although no studies have directly assessed the impact of undersized grafts in isolated tubular ascending aorta replacement, evidence from valve-sparing procedures suggests that excessive reduction of the sinotubular diameter can impair native valve function. Specifically, narrowing of the STJ results in an increased systolic transvalvular gradient. In the study by Reil et al., a sinotubular-to-annulus ratio below 1.2 was associated with a significant rise in mean transvalvular gradient, reaching up to 18 mmHg postoperatively, compared to less than 10 mmHg when the ratio was maintained above 1.3. This elevated gradient reflects increased left ventricular outflow resistance, which may compromise cardiac performance during exertion and increase the risk of aortic regurgitation or cusp prolapse [71]. The deleterious effects of this altered hemodynamic environment are also highlighted by Spadaccio et al. The persistent biomechanical mismatch between the rigid prosthesis and the native aortic tissue has been associated with a significantly higher incidence of postoperative complications. These include para-anastomotic aneurysms occurring in up to 10–15% of cases, left ventricular hypertrophy observed in 25–40% of patients, and the development or worsening of aortic insufficiency in 15–20%. In addition, chronic mechanical stress and local inflammatory responses triggered by the graft material may promote intimal hyperplasia and maladaptive aortic remodeling, ultimately increasing the risk of reintervention by approximately 12–18% [72].

Furthermore, several anastomotic techniques have been developed to optimize hemostasis and long-term aortic stability. Among them, end-to-end anastomosis remains the standard approach, providing simplicity and efficiency, but is sometimes associated with suture line bleeding (5–8% of cases), para-anastomotic pseudoaneurysms occurring in up to 10–15% of patients, or late dilatation at the STJ if not adequately reinforced (10–20% of cases). Anastomotic leaks, although less frequent (1–3%), may also present postoperatively and require careful imaging surveillance [73]. To address these limitations, alternative strategies have emerged. The telescopic anastomosis, in which the prosthetic graft overlaps and is parachuted inside the native aorta using interrupted pledgeted sutures, has been associated with improved mid-term outcomes. In a study including 120 patients, this technique demonstrated a significantly lower rate of STJ dilatation, with only 10% of patients experiencing significant dilation (>10 mm) at mid-term follow-up, compared to 30% in the conventional end-to-end group (*p* < 0.01). Moreover, the incidence of moderate to severe aortic insufficiency was reduced to 8% versus 20% in the standard technique group (*p* = 0.03). Freedom from reintervention at 3 years reached 95% with the telescopic method, against 85% with the classical approach, indicating superior long-term aortic stability and valve function preservation [74]. In addition, reinforcement methods such as the pericardial sandwich technique, where autologous pericardial strips are placed inside and outside the anastomosis, have proven effective in reducing bleeding, particularly in fragile tissues or coagulopathic patients, with postoperative bleeding rates reduced from around 11% to approximately 3%, and a significant decrease in transfusion requirements, all without increasing operative complexity or prolonging operative time [75]. More recently, adventitial eversion techniques have been developed to reinforce the proximal anastomosis in ascending aorta replacement. This method consists of folding the native aortic adventitia outward and everting the end of the prosthetic graft inward, creating a double-layered suture line that enhances mechanical strength and hemostasis. In the modified technique described by Zheng et al., interrupted sutures secure both the everted graft and adventitia together, forming a tight, reinforced junction. This approach demonstrated a significant reduction in operative time by about 20 min compared to conventional techniques (mean operative time: 120 vs. 140 min, *p* < 0.05). Furthermore, transfusion requirements were significantly lowered, with the rate of patients needing blood transfusions decreasing from 35% to 15% (*p* < 0.01). At one-year follow-up, rates of anastomotic complications such as bleeding, pseudoaneurysm, and reinterventions remained low and comparable to other reinforcement methods, all below 3%. These results suggest that adventitial eversion provides an efficient and safe alternative for improving hemostasis and potentially reducing operative burden [76].

In addition, the physiological consequences of replacing the compliant native aorta with a rigid prosthetic graft must also be acknowledged. The significance of the Windkessel effect was demonstrated by Hori et al., who showed that its loss following surgical or endovascular aortic replacement is associated with a marked increase in pulse wave velocity (PWV), often rising by 20–30% postoperatively, and elevated pulse pressure, sometimes increasing by 10–15 mmHg. As PWV rises, the reflected wave from peripheral vessels returns earlier to the ascending aorta during systole, resulting in higher systolic pressure and lower diastolic pressure. This elevation in pulse pressure and systemic afterload has been linked to adverse cardiovascular, cerebrovascular, and renal outcomes, including a 15–25% increased risk of myocardial infarction, a 20–30% higher incidence of stroke, and an accelerated decline in renal function, measured by a 10–20% faster reduction in glomerular filtration rate [77]. The limitations of Dacron grafts have also been confirmed by a recent study by Thomas et al. In a cohort of 322 Marfan patients, 124 underwent elective root replacement and 198 did not. Over a median follow-up of 9.9 years, the frequency of type B aortic dissection was 21% in the root replacement group versus 4.2% in the no-surgery group (*p* < 0.001). Aortic-related mortality was 11% post-replacement compared to 3.5% in controls (*p* < 0.01), and distal aortic interventions were significantly more frequent in the operated group [78]. These observations raise the question of whether such findings merely reflect secondary biases—for instance, the fact that Marfan patients undergoing surgery were likely a higher-risk population at baseline—or if they instead point toward a pathophysiological mechanism—notably, the loss of the Windkessel effect, potentially redirecting hemodynamic energy toward the descending aorta.

### 7.3. Other Techniques and Future Innovations

As mentioned before, the management of borderline distal ascending aortic aneurysms remains a subject of debate. Some surgeons may advocate for an open distal anastomosis performed under deep hypothermic circulatory arrest (DHCA), generally after cooling to between 18 and 32 °C, with the exact target temperature depending on the surgeon’s preference, institutional protocols, and the ability to complete the distal anastomosis within a short time frame, with or without the use of antegrade cerebral perfusion (ACP). This approach allows temporary cessation of blood flow in a controlled setting and enables more complete resection of diseased tissue [67]. While DHCA facilitates extensive resection of aneurysmal tissue in ascending aortic surgery, its use is associated with several time-dependent risks, particularly when circulatory arrest exceeds 30 to 40 min. In such cases, neurological complications, including stroke, are observed in approximately 5 to 15% of patients, while postoperative cognitive dysfunction may affect up to 30%. The need for peripheral arterial cannulation in DHCA further introduces vascular risks, with arterial injury and embolic events occurring in 3–7% of cases. Additionally, deep hypothermia impairs coagulation, contributing to increased perioperative bleeding and transfusion requirements, reported in up to 25% of patients [73]. To mitigate the risks linked to prolonged DHCA, adjunctive cerebral protection strategies such as ACP and retrograde cerebral perfusion (RCP) have become increasingly common. These techniques enable longer durations of circulatory arrest while preserving cerebral function. ACP, which delivers oxygenated blood directly to the brain via the carotid arteries, has shown superior neuroprotective efficacy compared to RCP, which relies on venous retrograde flow. Clinical data indicate that ACP can reduce the incidence of permanent neurological deficits to below 7%, even with circulatory arrest durations exceeding 40 min. In contrast, RCP provides limited cerebral metabolic support and is associated with less favorable neurological outcomes [79].

In terms of the complications arising from the use of DHCA and traditional cross-clamp resection methods, Rukosujew et al. have introduced a novel approach using transversal arch clamping, which enables complete resection of distal ascending aortic aneurysms without requiring open distal anastomosis or DHCA. In this technique, the aortic cross-clamp is positioned transversely on the proximal arch, just distal to the brachiocephalic trunk, allowing safe exclusion of the aneurysmal segment while preserving cerebral perfusion. The procedure is performed under moderate hypothermia and avoids peripheral cannulation, thereby reducing ischemic burden and simplifying the operative strategy. In their clinical series, this method was associated with a low in-hospital mortality rate of 1.4% and a neurological complication rate below 3%, alongside improved hemodynamic congruence between the graft and the native aorta. Despite these advantages, the technique requires substantial surgical expertise and is contraindicated in cases involving extensive arch disease or acute aortic dissection. Further multicenter studies are necessary to validate its long-term outcomes. Nonetheless, transversal arch clamping represents a promising alternative that balances thorough aneurysmal resection with minimized perioperative risk [67].

Regarding elderly patients or those with substantial comorbidities, the surgical risk linked to conventional ascending aortic replacement can be markedly elevated. Reported in-hospital mortality rates rise significantly with age: 11.1% in patients aged 80 and above and 3.7% in those aged 75–79 [80]. In such cases, external wrapping of the ascending aorta using synthetic grafts may offer a less invasive alternative that aims to reduce procedural trauma while providing structural reinforcement to the aortic wall. The technique involves encasing the dilated ascending aorta with a prosthesis of predetermined diameter, resulting in immediate reduction in aortic diameter, decreased wall shear stress, and potentially lower risk of dissection or rupture. A systematic review and meta-analysis of 17 studies including 722 patients reported a low hospital mortality rate of 1.5%, late aortic-related mortality of 0.3%, significant redilation in 1.7%, and a reoperation rate of 1.8%, supporting the long-term safety and durability of this technique in cases of moderate aneurysmal dilation. In light of these outcomes, González-Santos and Arnáiz-García reassessed the role of wrapping and concluded that, despite a shift in surgical paradigms favoring full replacement, wrapping remains a viable conservative strategy in carefully selected patients, particularly those at elevated operative risk. Nevertheless, the lack of robust long-term randomized data and evolving surgical preferences suggest that this technique should be reserved for scenarios prioritizing minimal invasiveness and procedural simplicity [81].

In addition to external wrapping, reduction ascending aortoplasty represents another conservative alternative for patients with moderate ascending aortic dilatation, particularly when concomitant cardiac procedures are indicated. The technique consists of resecting a segment of the dilated aortic wall, followed by direct double-layer closure to restore a near-normal caliber. It can be performed without reinforcement or combined with external wrapping using a Dacron graft, which provides additional structural support and helps maintain the reduced diameter [82]. Several series have reported favorable mid-term outcomes. In the largest cohort of unsupported reduction ascending aortoplasty, Walker et al. reported a mean diameter reduction from 4.55 ± 0.43 cm to 3.53 ± 0.44 cm, with only a minor late increase to 3.68 ± 0.41 cm after a median follow-up of 32 months, corresponding to a mean redilatation of 0.17 ± 0.27 cm while preserving Windkessel function [82]. When combining linear plication with Dacron wrapping, Ozcan et al. achieved a reduction from 4.7 ± 0.5 cm to 3.6 ± 0.4 cm, with 6% of patients developing redilatation > 4.5 cm at 4-year follow-up [83]. Niclauss et al., in 45 patients undergoing externally reinforced reduction aortoplasty, reported stable diameters at 5 years, with a low reintervention rate of 4.4% [84], while Feindt et al. observed no significant redilatation over 3.5 years in 43 patients treated with reinforced aortoplasty [85]. These findings suggest that reduction ascending aortoplasty, particularly when reinforced, offers a safe and effective solution for moderate, non-root-involving aneurysms, with shorter operative times and preservation of aortic compliance compared with full ascending aortic replacement. Nonetheless, redilatation risk remains higher in younger patients, those with bicuspid aortic valves, or those with larger preoperative diameters, reinforcing the need for long-term imaging surveillance.

Moreover, to address complications related to the loss of the Windkessel effect, innovative approaches are emerging. In particular, the development of compliance-matching aortic grafts offers promising prospects. In an experimental swine model, Rovas et al. evaluated a novel graft designed to replicate the native aorta’s compliance. Their results demonstrated that implantation of this graft limited the postoperative increase in pulse wave velocity to only 5%, compared to a 25–30% rise typically observed with conventional rigid grafts. Additionally, pulse pressure elevation was minimized to under 3 mmHg, suggesting a significantly reduced hemodynamic burden. Histological analyses confirmed favorable tissue integration with minimal inflammatory response [86]. Supporting these findings, De Paulis et al. reported on a third-generation Dacron graft specifically engineered for ascending aortic replacement. This graft demonstrated a 25% improvement in compliance compared to earlier models, leading to reduced postoperative stiffness and better hemodynamic profiles. Clinically, patients implanted with this graft exhibited a 15–20% reduction in postoperative pulse pressure compared to previous graft generations, alongside a significant decrease in graft-related complications such as thrombosis and local inflammation by approximately 30% during early follow-up (6–12 months). Imaging and histological assessments confirmed improved tissue integration with neointimal formation observed in over 80% of cases, suggesting favorable biocompatibility and healing. These preliminary results reinforce the promise of next-generation prostheses in addressing the physiological challenges posed by traditional rigid grafts and improving long-term patient outcomes [87].

To reduce surgical trauma, less invasive strategies have been explored. Angerer et al. proposed a surgical approach using a partial upper sternotomy. In this retrospective study including 167 elective ascending aortic replacements, outcomes following partial sternotomy were compared with those of full median sternotomy. The results demonstrated comparable rates of postoperative stroke (3% vs. 6%) and in-hospital mortality (5% vs. 3%), suggesting similar safety profiles. However, the partial sternotomy group exhibited a higher rate of re-exploration for bleeding (11% vs. 3%) [88]. More recently, Hamiko et al. reported on a totally endoscopic 3D-assisted technique via right anterior mini-thoracotomy for isolated ascending aorta replacement. In a multicenter cohort of 44 patients, 21 underwent isolated ascending aorta replacement using this minimally invasive approach. The results showed no 30-day mortality, no reoperations for bleeding, and a median hospital stay of 7.8 days, suggesting that in experienced hands, this technique can offer a safe and effective alternative to sternotomy, with reduced surgical trauma. However, larger-scale studies are still needed to confirm the reproducibility and long-term outcomes of this approach [89].

In parallel, the past decade has seen growing interest in thoracic endovascular aortic repair (TEVAR) as a potential alternative to open surgery for ascending aortic aneurysms, particularly in high-risk candidates. However, this approach remains limited by anatomical and technical challenges specific to this segment of the aorta. A recent systematic review analyzed 105 cases of endovascular repair for non-dissecting ascending aortic pathologies, including aneurysms, pseudoaneurysms, and penetrating ulcers. The technical success rate was high (99.05%), with an early mortality rate of 2.86%. Nonetheless, complications such as endoleaks (10.48%) and strokes (5.71%) were reported [90]. The major limitations of TEVAR in the ascending aorta include the lack of devices specifically designed for this region, the proximity of supra-aortic branches, and high hemodynamic forces. Custom-made devices have shown promising results, but their use remains limited to selected cases [91]. Long-term data are scarce, but some studies suggest acceptable durability in well-selected patients. For instance, one study reported an overall survival rate of 66.6% at a median follow-up of 14.4 months, with no aorta-related deaths [92]. Although endovascular repair of the ascending aorta offers a less invasive option for certain high-risk patients, it requires careful patient selection, advanced technical expertise, and close monitoring due to potential complications and the lack of long-term data.

## 8. Conclusions

Ascending aortic aneurysms represent a condition at the intersection of multiple epidemiological, genetic, mechanical, and biological factors. This literature review highlights the limitations of current management paradigms, which are primarily based on aortic diameter, and underscores the urgent need to adopt a more integrative and personalized approach. From a pathophysiological perspective, recent advances have enhanced our understanding of the cellular and molecular mechanisms involved in wall degradation, particularly the central role of vascular smooth muscle cells, the extracellular matrix, inflammatory pathways, and genetic alterations. These findings pave the way for the development of biological and imaging markers that could better predict the risk of acute complications. In parallel, technological progress in imaging modalities (such as 4D flow MRI and PET/CT) and the introduction of new morphological parameters (including aortic length, volume, and stiffness) provide promising tools to refine risk stratification. However, their integration into clinical practice still requires large-scale prospective validation. Surgically, ascending aortic replacement is a well-established and relatively straightforward procedure, with consistently low perioperative mortality and complication rates when performed in experienced centers. While novel approaches are being developed, such as minimally invasive access, external wrapping, and endovascular repair, they have yet to demonstrate clear superiority over open surgery, particularly in terms of long-term outcomes. The main challenge in managing this condition lies not in the complexity of the procedure itself but rather in the current surgical thresholds dictated by guidelines, which remain overly dependent on aortic diameter and may fail to prevent a significant number of dissections and ruptures. There is therefore an urgent need to redefine surgical indications by incorporating more sensitive and specific indicators. In conclusion, the optimal management of ascending aortic aneurysms requires a paradigm shift: it is no longer sufficient to simply measure a diameter; rather, clinicians must integrate a broad array of clinical, anatomical, functional, and biological data into an individualized, precision medicine-based approach.

## Figures and Tables

**Table 1:** Genetic mutations implicated in hereditary syndromes associated with thoracic aorta aneurysm development.

	Gene	Full Name	Protein Type Involved	Inheritance Pattern	Pathophysiological Role	Estimated Frequency
Marfan syndrome	*FBN1*	Fibrillin-1	Extracellular matrix (ECM) glycoprotein	Autosomal dominant	Deficient ECM integrity; impaired TGF-β sequestration; leads to medial degeneration	~2–3% of all TAAs; ~75% of syndromic TAAs
	*ACTA2, MYH11, MYLK, PRKG1, MAT2A, MFAP5, FOXE3, THSD4*	Various (actin, myosin, kinases, ECM regulators)	Smooth muscle and ECM regulators	Autosomal dominant	Impaired smooth muscle contraction and ECM stability	Variable and rare
Loeys–Dietz syndrome (types 1–5)	*TGFBR1, TGFBR2, SMAD3, TGFB2, TGFB3*	TGF-β receptors, SMAD proteins, TGF-β isoforms	TGF-β signaling proteins	Autosomal dominant	Dysregulated TGF-β signaling; excessive ECM remodeling and medial degeneration	<1% of all TAAs
Ehlers–Danlos syndrome (type IV)	*COL3A1*	Type III collagen	Fibrillar collagen	Autosomal dominant	Fragile vasculature due to collagen defect; spontaneous dissection or rupture	<1% of all TAAs
Turner syndrome	45,X (monosomy X)	-	Chromosomal anomaly	Sporadic (X-linked monosomy)	Congenital aortic disease: bicuspid valve, coarctation, ascending dilation	Common among females with Turner syndrome
Shprintzen–Goldberg syndrome	*SKI*	SKI proto-oncogene	TGF-β pathway regulator	Autosomal dominant	Aortic aneurysm due to dysregulated TGF-β signaling	Extremely rare
Alport syndrome (X-linked)	*COL4A5*	Type IV collagen alpha-5 chain	Basement membrane collagen	X-linked recessive	Type IV collagen defect affecting vasculature and renal basement membranes	Rare; <1% of TAAs

**Table 2:** Genetic mutations implicated in hereditary non-syndromic thoracic aorta aneurysm.

Gene	Full Name	Protein Type Involved	Inheritance Pattern	Pathophysiological Role	Estimated Frequency
ACTA2	Actin alpha 2, smooth muscle	Smooth muscle contractile protein	Autosomal dominant	Impaired vascular smooth muscle contractility	~10–15%
MYH11	Myosin heavy chain 11	Smooth muscle myosin protein	Autosomal dominant	Defect in actin–myosin sliding; occasionally associated with coarctation	<5%
MYLK	Myosin light chain kinase	Kinase regulating muscle contraction	Autosomal dominant	Disrupts myosin phosphorylation, reducing medial tone	Rare (~1–2%)
PRKG1	Protein kinase, cGMP-dependent, type I	Kinase modulating vascular tone	Autosomal dominant	Gain-of-function mutation leading to excessive aortic wall relaxation	Very rare
LOX	Lysyl oxidase	Collagen/elastin cross-linking enzyme	Autosomal dominant	Compromises the mechanical integrity of the medial layer	Rare (~1–2%)

**Table 3:** Levels of evidence and surgical indications (guideline comparison).

Context/Condition	ACC/AHA 2022	EACTS/STS 2024	ESC 2024
Asymptomatic TAA, tricuspid valve	Surgery ≥5.5 cm (COR 1)	Surgery ≥5.5 cm (Class I, Level B)	Surgery ≥5.5 cm (Class I, Level B)
Asymptomatic, experienced center	Consider surgery ≥5.0 cm in experienced centers (COR 2a)	Consider ≥5.0 cm in selected patients with risk modifiers (Class IIa, Level C)	Consider ≥5.0 cm with risk modifiers (Class IIa)
Marfan syndrome	Surgery ≥5.0 cm (≥4.5 cm if risk factors) (COR 2a)	Same (Class IIa, Level C)	Same (Class IIa, Level C)
Loeys–Dietz	≥4.0–4.5 cm depending on variant (COR 2a)	Similar (Class IIa, Level C)	Similar (Class IIa, Level C)
Pregnancy planning for women with connective tissue disorder	Surgery ≥4.0 cm recommended (COR 2b)	Not specifically addressed	Not specifically addressed

**Table 4:** Imaging modalities: characteristics, pros/cons, and recommended follow-up.

Imaging Modality	Strengths	Limitations	ACC/AHA 2022 (Recommended Interval)	EACTS/STS 2024 (Recommended Interval)	ESC 2024 (Recommended Interval)
Transthoracic echocardiography	Bedside, inexpensive, no radiation	Limited acoustic window, incomplete visualization of distal ascending aorta	Every 6–12 months if diameter ≥ 4.0 cm; every 6–24 months if stable (COR 2a)	Surveillance intervals should be considered after 5 years based on an individual protocol (Class IIa, Level C)	Every 6–12 months if stable; every 6 if growth rate > 3 mm/y (Class IIa, Level C)
CT angiography	High spatial resolution, 3D reconstruction, reproducible	Radiation exposure, contrast nephrotoxicity, motion artefact	Same intervals as TTE (COR 2a)	Similar to TTE (Class IIa, Level C)	Same intervals as TTE (Class IIa, Level C)
MRI	No radiation, excellent for serial follow-up	Longer exam time, less available, contraindications (implants)	Same intervals as TTE (COR 2a)	Similar to TTE (Class IIa, Level C)	Same intervals as TTE (Class IIa, Level C)

## Data Availability

Not applicable.

## Abbreviations

The following abbreviations are used in this manuscript:

BAV	Bicuspid Aortic Valve
CPB	Cardiopulmonary Bypass
DHCA	Deep Hypothermic Circulatory Arrest
ECM	Extracellular Matrix
HTAD	Heritable Thoracic Aortic Disease
PWV	Pulse Wave Velocity
RRT	Relative Residence Time
STJ	Sinotubular Junction
SUVmax	Maximum Standardized Uptake Value
TAA	Thoracic Aortic Aneurysm
TAWSS	Time-Averaged Wall Shear Stress
TEVAR	Thoracic Endovascular Aortic Repair
vSMCs	Vascular Smooth Muscle Cells
WSS	Wall Shear Stress
